# Effects of Different Exercise Modes on the Urinary Metabolic Fingerprint of Men with and without Metabolic Syndrome

**DOI:** 10.3390/metabo7010005

**Published:** 2017-01-26

**Authors:** Aikaterina Siopi, Olga Deda, Vasiliki Manou, Spyros Kellis, Ioannis Kosmidis, Despina Komninou, Nikolaos Raikos, Kosmas Christoulas, Georgios A. Theodoridis, Vassilis Mougios

**Affiliations:** 1 School of Physical Education and Sport Science at Thessaloniki, Aristotle University of Thessaloniki, 54124 Thessaloniki, Greece; vmanou@phed.auth.gr (V.Ma.); kellis@phed.auth.gr (S.K.); ikosmidia@phed.auth.gr (I.K.); kchristo@phed.auth.gr (K.C.); mougios@auth.gr (V.Mo.); 2 School of Chemistry, Aristotle University of Thessaloniki, 54124 Thessaloniki, Greece; oliadmy@gmail.com (O.D.); gtheodor@chem.auth.gr (G.A.T.); 3 Department of Nutrition and Dietetics, Alexander Technological Educational Institute of Thessaloniki, 57400 Thessaloniki, Greece; komninoud@gmail.com; 4 Laboratory of Forensic Medicine and Toxicology, School of Medicine, Aristotle University of Thessaloniki, 54124 Thessaloniki, Greece; raikos@med.auth.gr

**Keywords:** metabolomics, urinary metabolites, metabolic syndrome, exercise mode

## Abstract

Exercise is important in the prevention and treatment of the metabolic syndrome (MetS), a cluster of risk factors that raises morbidity. Metabolomics can facilitate the optimization of exercise prescription. This study aimed to investigate whether the response of the human urinary metabolic fingerprint to exercise depends on the presence of MetS or exercise mode. Twenty-three sedentary men (MetS, *n* = 9, and Healthy, *n* = 14) completed four trials: resting, high-intensity interval exercise (HIIE), continuous moderate-intensity exercise (CME), and resistance exercise (RE). Urine samples were collected pre-exercise and at 2, 4, and 24 h for targeted analysis by liquid chromatography-mass spectrometry. Time exerted the strongest differentiating effect, followed by exercise mode and health status. The greatest changes were observed in the first post-exercise samples, with a gradual return to baseline at 24 h. RE caused the greatest responses overall, followed by HIIE, while CME had minimal effect. The metabolic fingerprints of the two groups were separated at 2 h, after HIIE and RE; and at 4 h, after HIIE, with evidence of blunted response to exercise in MetS. Our findings show diverse responses of the urinary metabolic fingerprint to different exercise modes in men with and without metabolic syndrome.

## 1. Introduction

Metabolic syndrome (MetS) is a cluster of inter-related cardiometabolic risk factors including visceral obesity, hyperglycemia, dyslipidemia, and hypertension [1]. MetS significantly increases the risk for type 2 diabetes, cardiovascular disease, liver disease, and all-cause mortality [1,2,3]. Along with obesity and sedentary lifestyle, the prevalence of MetS has increased to epidemic proportions worldwide [4]. Although the exact mechanisms underlying the pathophysiology of the MetS are not yet understood, insulin resistance seems to play a central role, affecting many major metabolic pathways [5]. Metabolic fingerprinting has been increasingly applied to study obesity-related diseases. Metabolites, such as branched chain amino acids (BCAAs) [6,7,8], aromatic amino acids [8,9], glutamine [10], glutamate [10], glycine [8,11], nicotinuric acid [12], α-hydroxybutyrate [13], acylcarnitines [14], lysophosphatidylcholines [11,15], and trimethylamine-*Ν*-oxide [16], have been associated with obesity, insulin resistance, diabetes, and cardiovascular disease.

Regular physical activity and exercise hold a primary role in the prevention and treatment of MetS, helping to lower the associated risks [17,18,19]. Different exercise modes, i.e., endurance exercise, high-intensity interval exercise, and resistance exercise, have all been found to increase insulin sensitivity and improve individual risk factors comprising MetS, although possibly through different mechanisms [18,20]. To our knowledge, there has been no direct comparison of the acute effects of the aforementioned exercise modes on the metabolic fingerprint. Metabolomic investigation of the effect of exercise can shed light on the underlying biochemistry, promoting the understanding of the body’s response to the perturbation caused by exercise [21,22,23], which could translate into personalized exercise prescription for individuals with cardiometabolic risk factors. Exercise metabolomic studies so far have investigated isolated aspects of MetS, such as obesity [24,25] and insulin sensitivity [26,27,28], but not MetS as a clinical entity.

The aim of the study was to investigate whether the response of the human urinary metabolic fingerprint to exercise depends on the occurrence of MetS or on the type of exercise performed. In particular, we aimed to compare the effects of three fundamentally different exercise modes, namely, high-intensity interval exercise (HIIE), continuous moderate-intensity exercise (CME), and resistance exercise (RE), on the urinary metabolic fingerprints of sedentary, middle-aged men with or without MetS through different time points. The choice of urine as biological matrix was based on it being under no homeostatic mechanisms, easily accessible, stable, and less complex than other biofluids; therefore, an ideal source of biomarkers [29].

## 2. Results

Characteristics of the participants are summarized in Table 1. The two groups (i.e., MetS and Healthy) differed significantly in all parameters used as criteria of the metabolic syndrome except for HDL cholesterol, in which the difference almost reached statistical significance (*p* = 0.055). Groups also differed significantly in visceral fat rating, total cholesterol, LDL cholesterol, $\dot{V}$O_2_max, and HOMA2-IR. All of these differences pointed to a healthier profile of the Healthy group. Regarding body composition measurements, the observed difference in visceral fat rating is expected as MetS is characterized by central adiposity. Ten metabolites, namely, creatine, lactose, lysine, methionine, nicotinamide, pyruvate, thymine, trimethylamine, tryptamine, and tryptophan, were significantly higher in MetS compared to Healthy at baseline.

There was no significant difference between the HIIE and CME trials in terms of total distance covered in the 36 min of exercise for either group (MetS, 3.18 ± 0.43 vs. 3.14 ± 0.50 km, respectively; Healthy: 3.26 (3.13–3.69) vs. 3.53 (3.20–3.78) km, respectively). There was also no significant difference between groups regarding exercise intensity in each of the two trials (HIIE, 88% ± 2% HRmax for MetS vs. 87% ± 2% HRmax for Healthy; CME, 63% ± 3% HRmax for MetS vs. 64% ± 4% HRmax for Healthy). As an external validation of the UPLC-MS/MS metabolomic analysis, we also measured the lactate concentration spectrophotometrically. The spectrophotometrically measured lactate concentration exhibited significant correlation with the lactate levels as determined by UPLC-MS/MS (rs = 0.597, *p* < 0.001).

**Figure 1:**
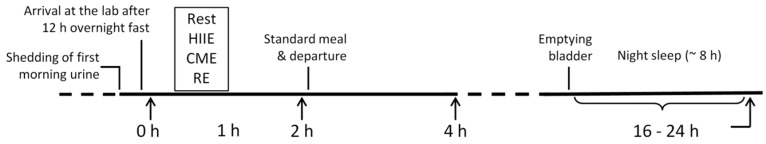
Overview of the experimental design. RE: resistance exercise, HIIE: high-intensity interval exercise, CME: continuous moderate-intensity exercise, REST: resting. Arrows indicate urine sampling.

Urine samples were collected in each exercise trial at baseline (set as 0 h), 2 h, 4 h, and 24 h (Figure 1). Urine samples were also collected at the same time points in the resting trial (REST), which served as the control trial.

### 2.1. Univariate Analysis

**Figure 2:**
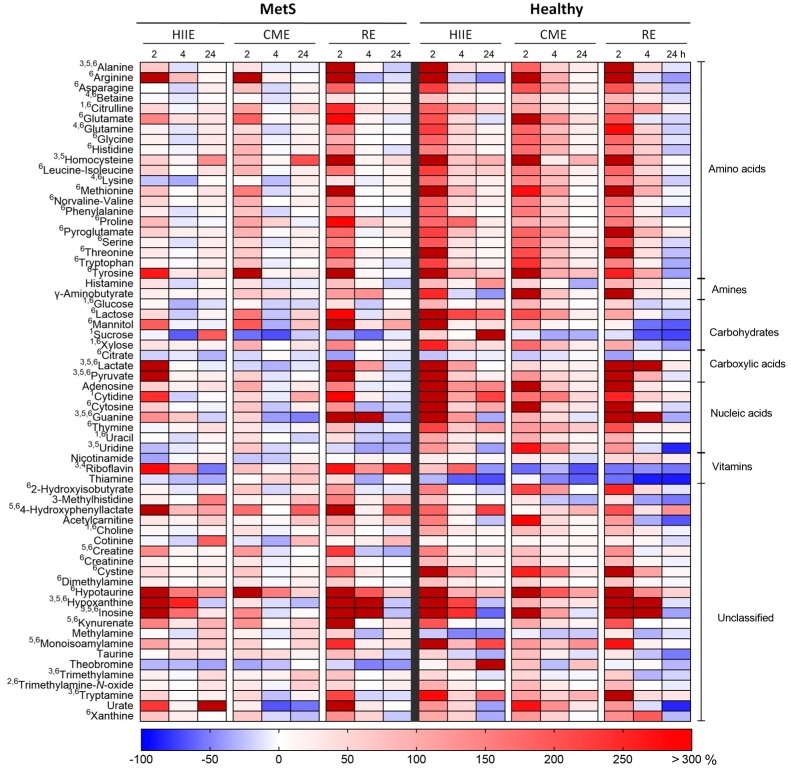
Heat map showing percent change from baseline in all measured metabolites at 2, 4 and 24 h for the three exercise modes in each group. Significant interactions (*p* < 0.05) from the three-way repeated-measure ANOVA are noted as follows: ^1^ exercise mode × group; ^2^ time × group; and ^3^ exercise mode × time. Significant main effects are noted as follows: ^4^ group; ^5^ exercise mode; and ^6^ time.

The results of the three-way ANOVA on all 64 identified metabolites are presented in the form of a heat map in Figure 2. No significant three-way interaction was observed, but two-way interactions were found in several metabolites: (i) The amino acid, citrulline; the monosaccharides, glucose, sucrose, and xylose; the nucleic acid components, cytidine and uracil; and choline presented significant interactions of exercise mode and group. (ii) Trimethylamine-*N*-oxide was the only metabolite to exhibit a significant time x group interaction, with groups being different at 2 and 24 h, MetS showing differences between 4 and 24 h, and Healthy showing differences between 2 h and all other time points. (iii) The amino acids, alanine and homocysteine; the purines, guanine, hypoxanthine, and inosine; the nucleoside, uridine; the carboxylic acids, lactate and pyruvate; the amines, trimethylamine and tryptamine; and riboflavin, presented significant exercise mode x time interactions. These interactions were mainly due to differences at the first post-exercise time point, where RE caused the highest increases. Hypoxanthine, inosine, and uridine also showed differences at the second and third post-exercise time points (mostly between HIIE and RE; and between CME and RE), with only hypoxanthine being different between HIIE and CME. 

**Figure 3:**
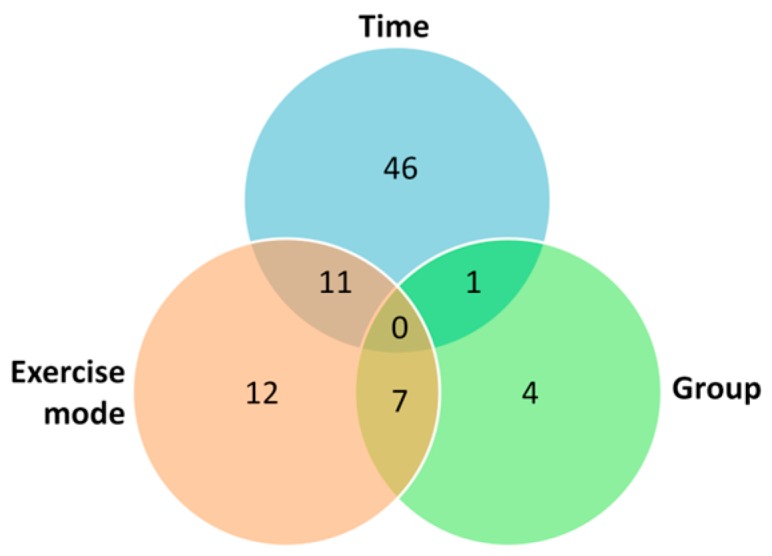
Venn diagram of the results of the three-way ANOVA. The numbers show the sums of significant main effects and interactions referring to all 64 metabolites determined by UPLC-MS/MS (*p* < 0.05).

Regarding main effects, glutamine, riboflavin, lysine, and betaine were found to differ significantly among groups. Twelve metabolites differed significantly among exercise modes. Ten of them differed significantly between CME and RE; seven differed between HIIE and CME; and five differed between HIIE and RE. Forty-six metabolites changed significantly over time. The effect of time was predominantly located in the comparison of the first post-exercise sample (2 h) with all other samples. Additionally, hypoxanthine, inosine, and xanthine were significantly different between 0 and 4 h; and alanine, guanine, and hypoxanthine were different between 4 and 24 h. The numbers of significant main effects and interactions referring to all 64 metabolites are summarized as a Venn diagram in Figure 3.

### 2.2. Multivariate Analysis

**Figure 4:**
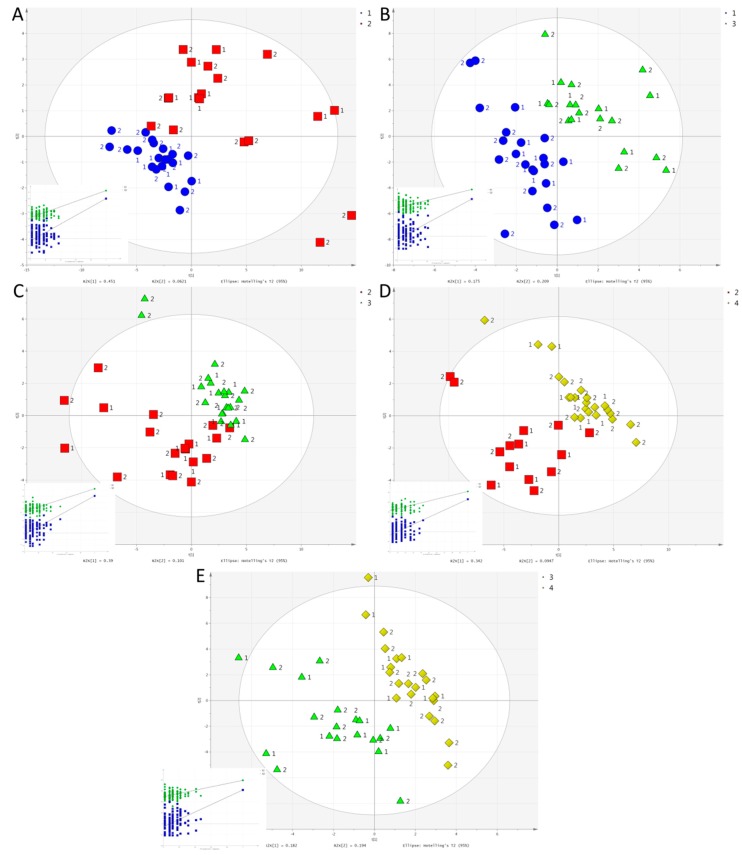
Score plots for the PLS-DA models of pair wise comparisons for RE: (**A**) 0 h (blue circles) vs. 2 h (red squares); (**B**) 0 h (blue circles) vs. 4 h (green triangles); (**C**) 2 h (red squares) vs. 4 h (green triangles); (**D**) 2 h (red squares) vs. 24 h (yellow diamonds); and (**E**) 4 h (green triangles) vs. 24 h (yellow diamonds). Inserts are permutation plots. The MetS group is represented as 1 and the Healthy group as 2. R2X (cum), R2Y (cum), Q2Y (cum), and CV-ANOVA *p* value: (**A**) 0.513, 0.724, 0.603, and 5.93 × 10^−7^; (**B**) 0.383, 0.778, 0.619, and 3.36 × 10^−6^; (**C**) 0.491, 0.720, 0.604, and 5.18 × 10^−7^; (**D**) 0.437, 0.819, 0.660, and 6.04 × 10^−7^; and (**E**) 0.376, 0.793, 0.617, and 2.76 × 10^−6^, respectively.

When group, exercise mode, and time were each set as the Y variable for pair wise PLS-DA, 16 out of the 39 possible comparisons produced valid models: (i) The two groups were separated at 2 h in HIIE and RE, as well as at 4 h in HIIE. (ii) Separation by exercise mode was achieved at 2 h between HIIE and CME; and between CME and RE. Separation was also achieved at 4 h between HIIE and RE; and between CME and RE. (iii) Separation by time was achieved in HIIE between 0 and 2 h; 0 and 4 h; 2 and 4 h; and 2 and 24 h. No valid models were constructed for CME. In RE, all time points were significantly separated except in the case of 0 vs. 24 h. Score plots for the pair wise comparisons of the different time points in RE are shown in Figure 4. The remaining valid models can be found in Supplementary Figures S1–S3. Table 2 shows the metabolites that were important in explaining the differences in each valid PLS-DA model according to their VIP scores.

Additionally, we compared exercise modes in each group individually. Separation was achieved between CME and RE at 2 h in both MetS and Healthy, between HIIE and CME at 2 h in Healthy, and between CME and RE at 4 h in Healthy (Supplementary Figure S4). Discriminating metabolites of these four models are presented in Supplementary Table S1.

## 3. Discussion

By applying a UPLC-MS/MS analytical method, we studied changes in the human urinary metabolic fingerprint under the influence of three independent factors: presence or not of MetS, exercise mode, and time relative to exercise. After performing both univariate (ANOVA) and multivariate (PLS-DA) statistical analysis, we found that time exerted the strongest differentiating effect on the metabolic fingerprint, followed by exercise mode and health status. We also found evidence for diminished metabolic flexibility of MetS in response to exercise. Of the three exercise modes, RE caused the greatest responses, followed by HIIE, while CME had minimal effect. Regarding the effect of time, the greatest changes were observed in the first post-exercise samples, with a gradual return to baseline at 24 h.

Groups differed significantly in maximal aerobic capacity ($\dot{V}$O_2_max), but this was expected, since aerobic capacity tends to be lower in obesity and diabetes. In fact, lean body aerobic capacity was significantly lower in sedentary men with MetS, compared to controls with matched levels of physical activity [30]. A delimitation of our study, then, was that participants exercised at the same relative, rather than absolute, intensity, which we deemed a “fairer” comparison. Had we chosen the same absolute intensity for both groups, the exercise stimulus could have been too low for the Healthy or too high for the MetS group, considering that the latter consisted of sedentary obese middle-aged men with cardiovascular risk factors and, hence, high chances of suffering injury or not completing the trials.

Multivariate statistical analysis distinguished groups after HIIE and RE. In particular, 3-methylhistidine, a marker of muscle protein turnover [31], showed higher relative increases after HIIE and RE, compared to REST, in Healthy than in MetS. However, in absolute terms, MetS had numerically higher values of 3-methylhistidine to begin with, possibly accounting for the observed differences in relative changes. Urinary 3-methylhistidine has been strongly associated with BMI [32]. Moreover, studies have failed to show an increase of interstitial 3-methylhistidine after strenuous aerobic or resistance exercise [33,34]. The higher increase of the amino acids, lysine, glutamine, and threonine, after HIIE in Healthy could point to higher protein degradation in general compared to MetS. The higher increase of acetylcarnitine in Healthy compared to MetS at 4 h after HIIE suggests higher carnitine *O*-acetyltransferase (CrAT) activity in response to exercise. CrAT catalyzes the reversible conversion of acetyl CoA and carnitine to acetylcarnitine and CoA in the mitochondrial matrix. Thus, CrAT is considered to serve in buffering the matrix acetyl CoA concentration, a pivotal metabolic parameter that increases during exercise as a result of increased carbohydrate and fatty acid catabolism. In fact, CrAT activity, known to regulate substrate switching and glucose tolerance [35], has been found diminished in obesity and diabetes [36]. On the other hand, the higher increase in hypoxanthine in MetS after HIIE suggests higher purine nucleotide degradation or less efficient hypoxanthine salvage process (by hypoxanthine-guanine phosphoribosyl transferase) compared to Healthy. 

When looking at the metabolite changes as a whole on the heat map in Figure 2, one concludes that Healthy experienced a more profound response to exercise than MetS. The main reason for this could be the metabolic inflexibility that characterizes obesity and MetS. Metabolic inflexibility is defined as the blunted capacity of skeletal muscle to switch between fat and carbohydrate utilization during fasting and postprandial conditions [37]. Indeed, obese older adults with impaired glucose tolerance exhibited diminished transition to carbohydrate oxidation during submaximal exercise than controls with normal glucose tolerance [38]. This could explain why MetS exhibited smaller changes after HIIE and CME than Healthy. Another reason for the blunted response of MetS to exercise could be impaired intramyocellular lipid oxidation. In sedentary men with MetS, lean body aerobic capacity, which was lower compared to controls with matched levels of physical activity, was inversely correlated with high-energy phosphate metabolism in skeletal muscle during exercise and with intramyocellular lipid content, attesting to diminished intramyocellular lipid oxidation [30]. Lastly, exercise training resulted in fewer adaptations in healthy relatives of type 2 diabetic patients than it did in healthy individuals with no family history of diabetes, suggesting that the genetic background could affect the response to exercise [39].

Regarding the comparison of the three exercise modes employed in the present study, it is apparent that RE had the most profound and sustained effect on the urinary metabolic fingerprint, at least as determined with the particular method that mostly identifies polar compounds. HIIE had a lower impact, leaving CME with the weakest effect. As far as dependency on energy systems is concerned, the three exercise modes chosen cover the full spectrum of energy systems, ranging from predominance of the aerobic system in CME to a considerable contribution of the lactate system in HIIE to predominance of the anaerobic systems (lactate and ATP-phosphocreatine) in RE [40]. Therefore, it is expected that different exercise modes will result in different responses of the metabolic fingerprint. 

A related exercise metabolomic study on well-trained athletes showed distinct differences in the plasma metabolic fingerprint between HIIE and CME, with exercise intensity-dependent changes in tricarboxylic acid cycle intermediates, lactate, alanine, glutamate, tyrosine, and specific nonesterified fatty acids [41]. In another exercise study, performed on healthy untrained men, HIIE caused higher plasma lactate and hypoxanthine concentrations, as well as higher urinary excretion of hypoxanthine and urate, compared to work-matched CME [42]. The authors suggested that higher urinary purine excretion after HIIE was a result of net loss of ATP from the muscle. During short, intense, intermittent efforts, as those in the RE and HIIE trials, there is an acute degradation of AMP in the skeletal muscle through a series of reactions catalyzed by AMP deaminase, 5′-nucleotidase and purine nucleoside phosphorylase, which produce inosine monophosphate, inosine and hypoxanthine, respectively. This is in accordance with results from the present study, where higher increases in hypoxanthine and inosine discriminated HIIE and RE from CME at 2 h, as well as RE from CME at 4 h. Moreover, at 4 h, hypoxanthine discriminated RE from HIIE (exhibiting a higher increase in the former). Higher lactate and pyruvate increases discriminated HIIE and RE from CME at 2 h. The higher increase in alanine after RE compared to CME can be explained by the conversion of pyruvate to alanine through the activity of alanine aminotransferase. Higher increases in urinary guanine and hypoxanthine after RE compared to CME also point to higher purine nucleotide degradation and a possible saturation of the purine salvage process by hypoxanthine-guanine phosphoribosyl transferase. All of these observations confirm the higher dependence of RE on the ATP-phosphocreatine and lactate systems for energy production, compared to HIIE and, even more, CME. On the other hand, uridine exhibited higher increases after CME compared to HIIE or RE, while it also discriminated HIIE from RE at 4 h (exhibiting a higher increase in the former). Uridine is a pyrimidine nucleoside that has been found to increase in plasma after exercise when there is high ATP degradation and increased UDP-glucose consumption for glycogen synthesis [43]. As both of these processes would be expected to be augmented more after HIIE and RE than after CME, our finding seems counterintuitive and we cannot explain it. 

It is known that the impact of exercise on metabolism carries on for several hours after the cessation of exercise and is characterized by increased lipid oxidation and glycogen resynthesis [44]. In the present study, most of the metabolites studied changed significantly after exercise. As expected, the greatest changes were observed in the first post-exercise samples, and then there was a gradual return to baseline at 24 h, when no significant differences from pre-exercise were observed. Changes indicate increased degradation of purines, carbohydrates, proteins, and amino acids with exercise. These results are in accordance with previous work from our group and others on the changes of the urinary metabolic fingerprint after acute exercise [45,46]. However, the present study included individuals with MetS, in addition to healthy non-athletes, three different exercise modes, and a 24 h follow-up. There were also changes in methylamines (dimethylamine, trimethylamine, and trimethylamine-*N*-oxide), which are produced by bacterial degradation of choline and carnitine in the gut. These changes could be related to increased blood circulation in the gut after exercise, resulting in increased absorption of these metabolites into the enterohepatic system, or to other unknown factors. 

## 4. Materials and Methods

### 4.1. Participants

Twenty-three sedentary men (defined as performing ≤ 2 h/week of physical exercise) were divided into two groups: MetS (*n* = 9) and Healthy (*n* = 14). Assignment to the MetS group was according to the National Heart, Lung, and Blood Institute/American Heart Association criteria, that is, presence of at least three of the following: central obesity (waist circumference ≥ 102 cm), high circulating fasting triacylglycerols (≥150 mg/dL, or 1.7 mmol/L), low HDL cholesterol (<40 mg/dL, or 1.0 mmol/L), high fasting glucose (≥100 mg/dL, or 5.6 mmol/L), and high blood pressure (systolic ≥ 130 mm Hg and/or diastolic ≥ 85 mm Hg) [47]. Exclusion criteria were the absence of sedentary lifestyle, any contraindication for exercising, acute or chronic disease, use of medication or dietary supplements, smoking, and dieting or recent change in body weight (>2 kg within the past 6 months). All participants provided written informed consent before entering the study, which was approved by the institutional ethics committee and was conducted in accordance with the Helsinki declaration of 1975, as revised in 2008. 

### 4.2. Preliminary Testing

One to two weeks before the onset of the study, all participants were subjected to preliminary tests that included anthropometric measurements (weight, height, waist circumference, and body fat), biochemical screening (fasting serum glucose, insulin, triacylglycerols, total cholesterol, HDL cholesterol, and LDL cholesterol), and blood pressure measurements. Additionally, they filled out medical history, dietary, and physical activity questionnaires. Biochemical analysis was performed in an automatic analyzer (Cobas Integra 400 plus, Roche Diagnostics, Basel, Switzerland). Insulin was measured by enzyme immunoassay, using a kit from IBL International (Hamburg, Germany). Body fat was assessed by bioelectrical impedance analysis using the Bodystat 1500 (Bodystat, Douglas, UK). Trunk and visceral fat were assessed by bioelectrical impedance analysis using the Tanita Viscan AB140 (Tanita, Tokyo, Japan). Maximal oxygen uptake ($\dot{V}$O_2_max) and maximal heart rate (HRmax) were determined through an incremental exercise test to exhaustion, as described [48]. Muscular strength, expressed as one-repetition maximum (1 RM), was predicted after 7–10 RM tests, since it is not recommended for novice lifters to perform 1 RM tests (because of increased risk for injury) [49].

### 4.3. Experimental Protocol

Using a crossover design, all participants completed four trials: resting (REST), which served as the control trial, one bout of high-intensity interval exercise (HIIE), one bout of continuous moderate-intensity exercise (CME), and one bout of resistance exercise (RE). Each participant completed the trials in random order. (A random-number generator software was used to provide random sequences.) Trials for each participant were spaced 5 to 7 days apart. 

Participants were instructed to keep records of their physical activity for two days before each trial and the day of the trial. They were asked to refrain from exercise during this period and carry out only the activities of daily living. Their physical activity levels for those days were verified with the use of a pedometer (PD-637, Tanita, Tokyo, Japan). Participants were also instructed to keep records of their diet and avoid alcohol and caffeine consumption during the same period. They were further instructed to replicate the diet of the two days preceding the first trial on the two days preceding the next trials (i.e., purchase the same type and brand of foods, use the same cooking methods and portions, etc.) in order to ensure the same pre-trial energy and nutrient intakes. On the day before and the day of each trial, participants followed a prescribed diet, designed to meet their individual energy needs and provide 50% of energy from carbohydrate, 20% from protein and 30% from fat. Individual energy needs were estimated by multiplying resting energy expenditure (calculated using the Harris–Benedict equation [50]), by an activity factor of 1.5 reflecting the light physical activity levels of the participants [51]. 

On the day of each trial, participants arrived at the lab in the morning after 12 h of overnight fast and after they had emptied their bladders of the first morning urine. They were weighed, a 24-h dietary recall was obtained, and their dietary and physical activity records were examined. During this time participants remained seated to relax. Then, they provided baseline urine samples and either performed exercise in the HIIE, CME, and RE trials or remained seated and relaxed in the REST trial.

In the HIIE trial, participants walked and/or ran on a treadmill (Technogym Runrace, Gambettola, Italy). After 4 min of warm-up at 3 km/h, they alternated four times between 4 min at 90% of HRmax and 4 min at 3 km/h. Thus, total exercise time was 36 min, 16 min of which was at high intensity. Heart rate was monitored continuously by a telemetric monitor (F1-90440, Polar Electro Oy, Kempele, Finland). Speed was adjusted as necessary to maintain 90% of HRmax during the high-intensity segments. In the CME trial, participants walked or ran continuously at 65% of HRmax for 36 min. Heart rate was monitored continuously in the same way as above. In the RE trial, participants performed 6 types of resistance exercises (leg extension, chest press, leg curl, lat pull down, leg press, and biceps curl) designed to target all major muscle groups. Participants completed 3 sets of 8–12 repetitions at 75%–80% of 1RM for each exercise. The duration of each set was approximately 2 min, and the total duration of the trial was 45 min. The three exercise trials were timed so as to end one hour after the baseline urine collection (Figure 1).

### 4.4. Sample Collection and Preparation 

Urine samples were collected in each trial at baseline (set as 0 h), 2 h, 4 h, and 24 h. During exercise and the ensuing one hour, participants were given only water to drink. They were instructed to mark their water consumption during the first trial and replicate it during the rest of the trials. Following urine sampling at 2 h, they consumed a standard mixed breakfast and then left the lab. They were instructed not to eat or drink anything but water for the next 2 h and to collect their urine at 4 h, as well as their first morning urine (after 12 h of overnight fast) at 24 h, along with any night urine between 16 and 24 h (Figure 1). Participants were asked to empty their bladders in all cases. Samples were kept refrigerated during this period. In the end, each sample was mixed well, three 1.5-mL aliquots were stored at −80 °C, and the rest was discarded.

Samples were thawed and mixed just prior to processing. Then 300 μL of each sample were diluted with 600 μL of cold acetonitrile (−24 °C), mixed for 20 min and centrifuged at 14,573× *g* for 18 min. The supernatant was passed through polytetrafluoroethylene (PTFE) filters for high-performance liquid chromatography (0.22 μm pore diameter), and ~90 μL of the filtrate were placed in appropriate glass inserts within LC-MS vials, which were in turn placed in the refrigerated (4 °C) autosampler of the liquid chromatograph for analysis. 

### 4.5. UPLS-MS/MS Analysis

Samples were analyzed through a targeted hydrophilic-interaction ultraperformance liquid chromatography–tandem mass spectrometry (HILIC-UPLC-MS/MS) method, as previously described [52], on an ACQUITY liquid chromatograph in tandem with a Xevo TQD MS System (Waters, Milford, MA, USA), equipped with the MassLynx software, using an ACQUITY HILIC, BEH amide column (2.1 mm × 150 mm, 1.7 μm) at 40 °C. A gradient elution of solvent A (acetonitrile-water 95:5 (*v*/*v*)) versus solvent B (acetonitrile-water 30:70 (*v*/*v*), 0.01% ammonium formate) was used at a flow rate of 0.5 mL/min, and the sample volume injected was 5 μL. A polarity switching mode (both positive and negative) of electrospray ionization was applied. The capillary voltage was +3.5 KV, while the voltage of the cone and the collision energy were adjusted for each analyte as described [52], where chromatographic and mass spectrometric characteristics of each identified metabolite are reported. Block and desolvation temperatures were 150 and 350 °C, respectively. The total analysis time was 40 min. A mix of standards of the targeted metabolites at three concentration levels was used as a quality control sample and was periodically injected to confirm the stability of the analytical system. Urine samples were analyzed spectrophotometrically for lactate as described [21].

### 4.6. Data Handling and Statistical Analysis

Insulin resistance was assessed using the HOMA Calculator v2.2.3 (Diabetes Trials Unit, The Oxford Center for Diabetes, Endocrinology and Metabolism, Oxford, UK) to calculate the HOMA2-IR score [53]. For each metabolite, the mean of the values at 0 h in the four trials was used to compare baseline levels between groups. Data are expressed as the mean ± SD or median (interquartile range), depending on whether their distribution does not differ or does differ significantly from normal, respectively, as judged by the Shapiro–Wilk test. Differences between groups in anthropometric characteristics, criteria of the MetS, HOMA-IR, baseline metabolites levels, and exercise intensity (%HRmax, in the HIIE and CME trials), were examined with Student’s *t* test for normally distributed data or Mann–Whitney U test for not normally distributed data. Total distance covered in the HIIE and CME trials was compared within each group with paired-sample *t* test for normally distributed data or Wilcoxon signed-rank test for not normally distributed data. Lactate levels determined by UPLC-MS/MS were correlated with those determined spectrophotometrically using Spearman’s correlation. UPLC-MS/MS data were processed with the TargetLynx software (Waters, Milford, MA, USA). For the results of the metabolomic analysis, the peak area of each metabolite in the samples of the exercise trials was normalized to the respective value in the REST trial to correct for batch effect and to isolate the effects of exercise from other confounding factors and effects such as feeding, diurnal variation, and possible glomerular hyperfiltration associated with obesity or pre-diabetes [54]. A 2 (group) × 3 (exercise mode) × 4 (time) ANOVA with repeated measures on exercise mode and time was performed on the 64 identified metabolites. Significant main effects of exercise mode and time were followed up with post-hoc tests using Sidak’s adjustment for multiple comparisons, whereas significant interactions were followed up with simple main effects analysis using Sidak’s adjustment too. Univariate statistical analysis was performed using SPSS, v. 22 (IBM, Chicago, IL, USA), and *p* values below 0.05 were considered significant. 

Multivariate statistical analysis was performed on SIMCA 13.0 (Umetrics, Umea, Sweden). Peak areas were subjected to univariate scaling and analyzed with principal component analysis (PCA) and partial least square discriminant analysis (PLS-DA), followed by validation of permutations to check the validity of the generated models (100–150 permutations, proportional to the number of samples). Observations exceeding 99% of the Hotelling’s T2 tolerance region in the PCA score plots were considered outliers and were excluded. Metabolites were considered important in explaining separation in each model based on the respective loadings plots and a Variable Importance on Projection (VIP) score above one.

## 5. Conclusions

We monitored the changes of 64 urinary metabolites for 24 h after acute bouts of three exercise modes in men with and without MetS. Our findings show that exercise caused profound metabolic perturbations, which persisted for 3 h after RE and HIIE, but subsided the next day. To our knowledge, the metabolic fingerprints of three fundamentally different exercise modes were compared for the first time, and the differences found reflect the differences in the energy systems that dominate in each exercise. The greatest response was observed after RE, followed by HIIE, while CME’s effect was weak. Finally, we found evidence of a blunted response to exercise in individuals with MetS compared to Healthy. Further investigation may bring us closer to personalized exercise prescription for individuals with cardiometabolic risk factors. Overall, our findings indicate changes in several metabolic pathways induced by different exercise modes and support the value of urinary metabolic fingerprinting in the study of exercise metabolism.

## Figures and Tables

**Table 1:** Characteristics of the participants at baseline.

	MetS (*n* = 9)	Healthy (*n* = 14)
Age (years)	46 ± 8	41 ± 7
Weight (kg)	100 ± 10	91 ± 15
Height (m)	1.80 ± 0.07	1.80 ± 0.07
BMI (kg·m^−2^)	31.0 ± 3.7	28.1 ± 4.2
Waist circumference (cm)	110 (103–117)	99 (94–108) *
Body fat (% body weight)	26.9 ± 5.5	22.7 ± 5.0
Trunk fat (% area)	37.7 ± 4.9	33.1 ± 7.3
Visceral fat rating	21 (15–24)	15 (12–19) *
Serum glucose (mmol·L^−1^)	5.9 (5.6–6.6)	5.2 (5.0–5.4) ***
Serum triacylglycerols (mmol·L^−1^)	1.9 (1.3–2.9)	1.1 (0.9–1.5) **
Serum total cholesterol (mmol·L^−1^)	6.4 ± 1.3	5.2 ± 0.8 **
HDL cholesterol (mmol·L^−1^)	1.2 ± 0.3	1.4 ± 0.3
LDL cholesterol (mmol·L^−1^)	4.2 ± 1.2	3.1 ± 0.8 *
Systolic pressure (mm·Hg)	140 ± 15	120 ± 9 ***
Diastolic pressure (mm·Hg)	87 (84–98)	76 (72–80) ***
$\dot{V}$O_2_max (mL·kg^−1^·min^−1^)	31.1 ± 4.2	37.0 ± 4.1 **
Resting heart rate (bpm)	67 ± 10	64 ± 9
HRmax (bpm)	176 ± 11	179 ± 13
HOMA2-IR	3.06 ± 1.04	2.22 ± 0.82 *

Data are mean ± SD or median (interquartile range). * *p* < 0.05, ** *p* < 0.01, *** *p* < 0.001: significantly different from MetS based on Student’s *t* test or Mann–Whitney U test, as appropriate.

**Table 2:** Important metabolites in explaining the valid PLS-DA models.

	Group			Exercise Mode				Time								
	HIIE, 2 h	HIIE, 4 h	RE, 2 h	2 h		4 h		HIIE				RE				
	MetS vs. Healthy	MetS vs. Healthy	MetS vs. Healthy	HIIE vs. CME	CME vs. RE	HIIE vs. RE	CME vs. RE	0 vs. 2 h	0 vs. 4 h	2 vs. 4 h	2 vs. 24 h	0 vs. 2 h	0 vs. 4 h	2 vs. 4 h	2 vs. 24 h	4 vs. 24 h
3-Methylhistidine	1.69 **	1.03 **	1.63													
4-Hydroxyphenyllactate				−0.49 *												
Acetylcarnitine		1.07 *														
Alanine					1.84 **					−0.58 ***	−0.61 ***	3.16 ***		−0.65 ***	−0.73 ***	
Arginine							−0.64 *						−0.35 *		−0.43 ***	0.32 *
Citrate					0.26 **							−0.35 ***		0.48 ***		
Creatine														−0.45 ***		
Creatinine								−0.53 ***								
Cystine																−0.25 *
Cytosine							−0.29									
Dimethylamine												0.51 ***			−0.22 ***	
Glucose								0.57 ***		−0.40 ***						
Glutamate							−0.30									
Glutamine	1.79 **											1.43 ***			−0.46 ***	
Guanine					1.31 ***			1.97 ***	0.91 *		−0.74 ***	2.69 ***			−0.77 ***	−0.76 *
Histamine																−0.26 **
Histidine																−0.29 *
Hypotaurine												2.14 ***				
Hypoxanthine	−0.39			−0.77 ***	10.21 ***	1.57	6.38 ***	7.01 ***	1.85 ***	−0.55 ***	−0.89 ***	16.78 ***	7.45 ***		−0.94 ***	−0.90 ***
Inosine				−0.71 ***	37.43 **		4.73 **		2.40 ***				7.28 ***			−0.91 ***
Kynurenate	0.81									−0.55 ***				−0.51 ***		
Lactate				−0.93 **	60.84 ***			21.59 ***		−0.92 **	−0.96 ***	58.70 ***	5.83	−0.87 ***	−0.98 ***	
Lysine	1.51 **	0.69 *														
Methylamine																0.65 **
Monoisoamylamine		0.68 *		−0.40 *		−0.36 ***						1.41 ***		−0.50 ***		
Proline								0.81 *								
Pyroglutamate								1.47 ***			−0.50 ***	1.85 ***				
Pyruvate				−0.75 ***	16.54 ***							18.25 ***		−0.90 ***	−0.94 ***	
Riboflavin	−0.81 *	−0.76	−0.69 *													
Serine												1.13 ***				
Sucrose													−0.72 *			
Thiamine						−0.58	−0.51 *									0.42
Threonine	1.70 **															
Thymine	1.88 **															
Trimethylamine														−0.44 ***		0.19
Uracil							−0.27 *									
Uridine				0.53	−0.41 *	−0.44 *	−0.43 **					−0.13	−0.39 *			
Xanthine								0.94 ***			−0.55 ***				−0.51 ***	−0.51 ***

Numbers indicate fold change and appear wherever a metabolite contributed to the discrimination. For example, the first number, 1.69, means that the value in Healthy was 1.69 fold higher than the value in MetS. * *p* < 0.05, ** *p* < 0.01, *** *p* < 0.001, significant difference following Student’s *t* test.

## Supplementary Materials

The following are available online at www.mdpi.com/2218-1989/7/1/5/s1, Table S1: Important metabolites in explaining PLS-DA models of the comparison of exercise modes in each group separately. Figure S1: Score plots for the valid PLS-DA models of MetS vs. Healthy. Figure S2: Score plots for the valid PLS-DA models of pair wise comparisons of exercise modes. Figure S3: Score plots for the valid PLS-DA models of pair wise comparisons of different time points for HIIE. Figure S4: Score plots for the valid PLS-DA models of pair wise comparisons of exercise modes in each group separately.

## Abbreviations

AMP	adenosine monophosphate
ANOVA	analysis of variance
ATP	adenosine triphosphate
BCAAs	branched-chain amino acids
BMI	body mass index
CME	continuous moderate-intensity exercise
CrAT	carnitine O-acetyltransferase
HDL	high-density lipoprotein
HIIE	high-intensity interval exercise
HILIC	hydrophilic interaction
HOMA-IR	homeostasis model assessment–insulin resistance
HRmax	maximal heart rate
LC-MS	liquid chromatography–mass spectrometry
LDL	low-density lipoprotein
MetS	metaboli syndrome
PLS-DA	partial least square discriminant analysis
PTFE	polytetrafluoroethylene
RE	resistance exercise
RM	repetition maximum
UDP	uridine diphosphate
UPLC-MS/MS	ultraperformance liquid chromatography–tandem mass spectrometry
$\dot{V}$O_2_max	maximal oxygen uptake
VIP	variable importance on projection
