# Supplementary material for: Effects of Different Exercise Modes on the Urinary Metabolic Fingerprint of Men with and without Metabolic Syndrome
Source: Metabolites. 2017 Jan 26;7(1):5. doi: 10.3390/metabo7010005 (PMC5372208; doi:10.3390/metabo7010005)
Supplement: Supplementary file 1 [file metabolites-07-00005-s001.pdf]

Supplementary Materials: Effects of Different Exercise Modes on the Urinary Metabolic Fingerprint of Men with and without Metabolic Syndrome

Aikaterina Siopi, Olga Deda, Vasiliki Manou, Spyros Kellis, Ioannis Kosmidis, Despina Komninou, Nikolaos Raikos, Kosmas Christoulas, Georgios A. Theodoridis and Vassilis Mougios

Table S1. Important metabolites in explaining the PLS-DA models of the comparison of exercise modes in each group separately.

	MetS	Healthy		
	2 h	2 h	2 h	4 h
	CME vs. RE	HIIE vs. CME	CME vs. RE	CME vs. RE
Alanine	2.60 **		1.57	
4-Hydroxyphenyllactate		−0.62 **		
Citrate	−0.37 *			
Creatine		−0.46 **	0.59	
Glutamate				−0.49 *
Guanine	2.10 ***		0.94	
Homocysteine	5.12 *			
Hypoxanthine	12.88 **	−0.75 ***	8.35 ***	6.45 **
Inosine			32.92 *	4.86 *
Lactate	92.88 **	−0.95 **	48.67 ***	
Monoisoamylamine			1.07 *	
Pyruvate	11.58 **		18.68 **	
Trimethylamine	0.81 **			
Tryptamine			1.15 *	
Uracil				−0.35 *
Uridine			−0.41 *	−0.50 *

Numbers indicate fold change and appear wherever a metabolite contributed to the discrimination. For example, the first number, 2.60, means that the value in RE was 2.60 fold higher than the value in CME. * $p < 0.05$, ** $p < 0.01$, *** $p < 0.001$, significant difference following Student's *t* test.

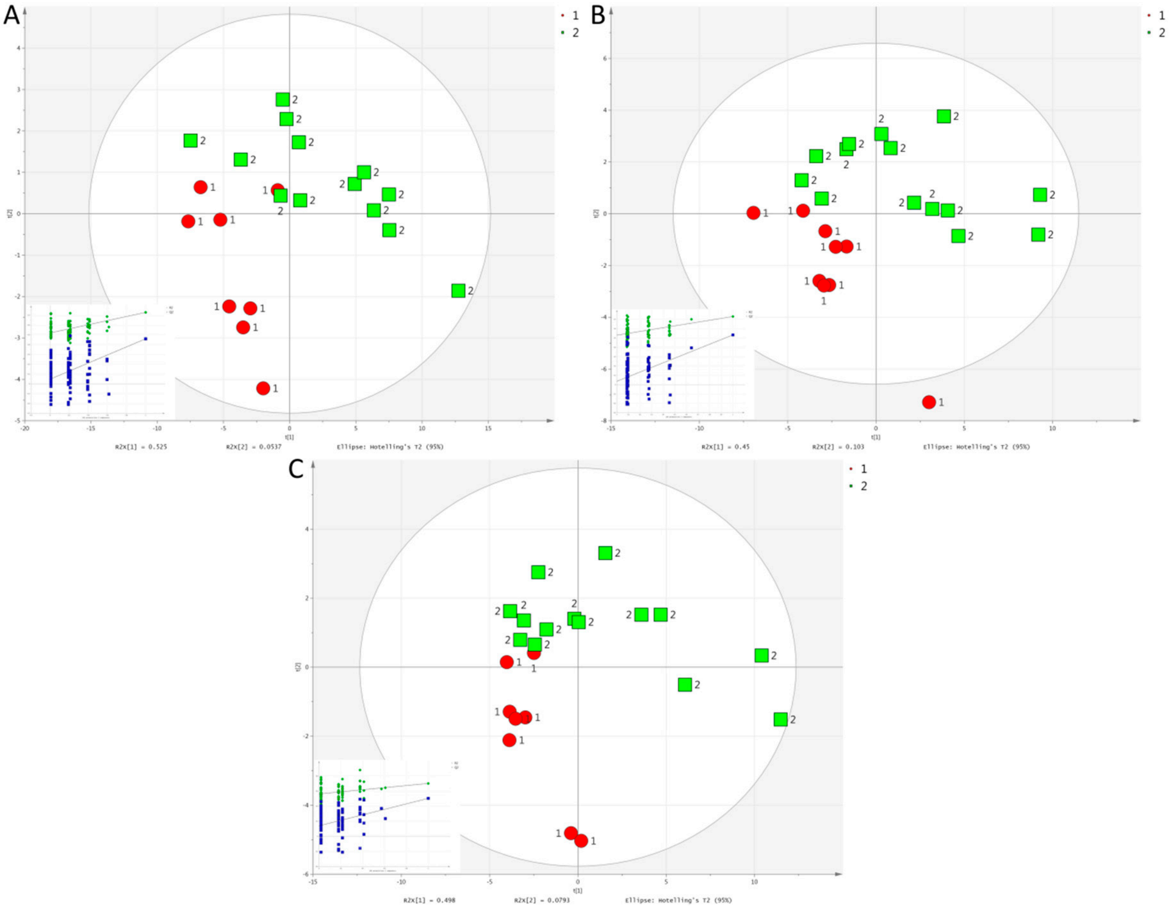

Figure S1. Score plots for the PLS-DA models of MetS (red circles) vs. Healthy (green squares) for: (**A**) HIIE at 2 h; (**B**) HIIE at 4 h; and (**C**) RE at 2 h. Inserts are permutation plots. R2X (cum), R2Y (cum), Q2Y (cum), and CV-ANOVA *p* value were: (**A**) 0.579, 0.723, 0.459, and 3.58×10^{-2}; (**B**) 0.554, 0.730, 0.533, and 5.84×10^{-3}; and (**C**) 0.577, 0.702, 0.504, and 2.46×10^{-2}, respectively.

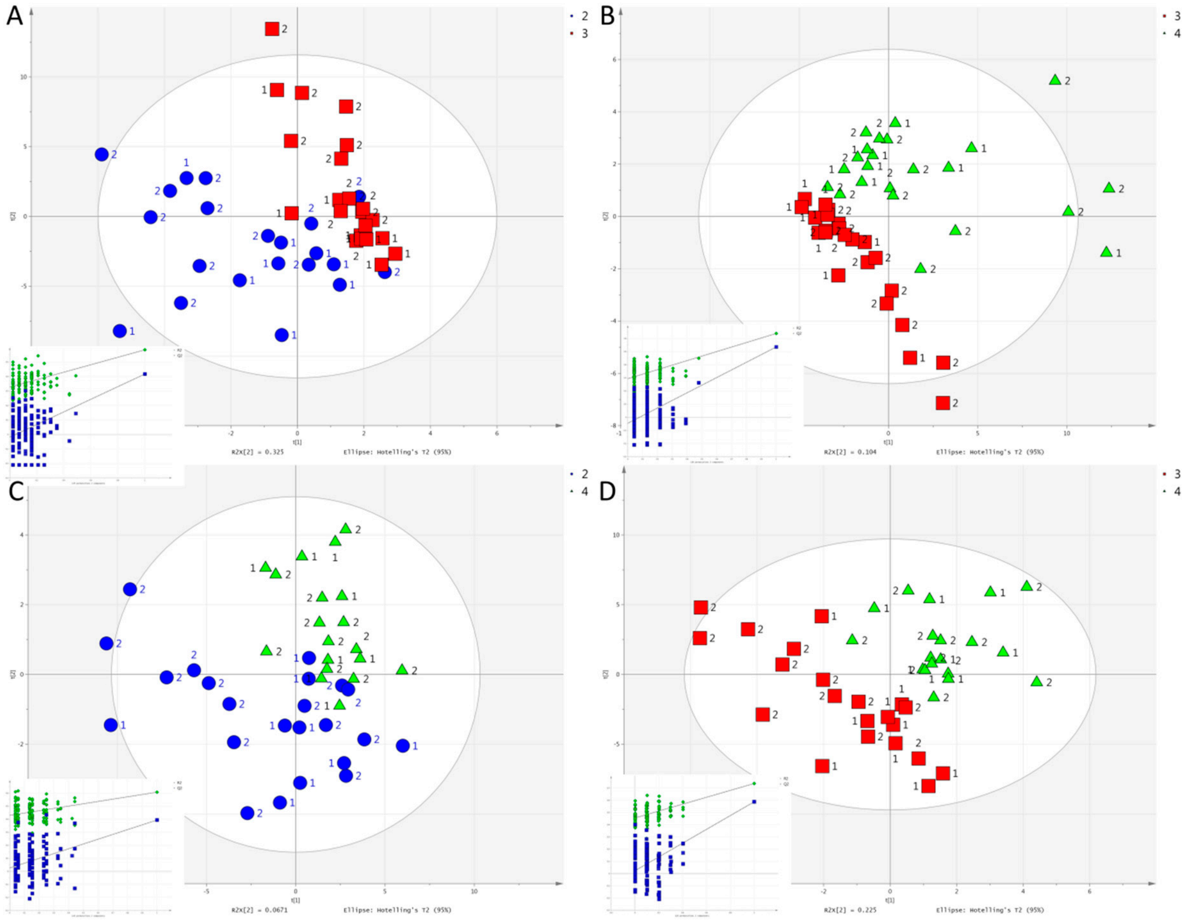

Figure S2. Score plots for the PLS-DA models of pair wise comparisons of exercise modes: (**A**) at 2 h HIIE (blue circles) vs. CME (red squares); (**B**) at 2 h CME (red squares) vs. RE (green triangles); (**C**) at 4 h HIIE (blue circles) vs. RE (green triangles); and (**D**) at 4 h CME (red squares) vs. RE (green triangles). Inserts are permutation plots. The MetS group is represented as 1 and the Healthy group as 2. R2X (cum), R2Y (cum), Q2Y (cum), and CV-ANOVA *p* value were: (**A**) 0.572, 0.584, 0.414, and 2.26×10^{-4}; (**B**) 0.570, 0.638, 0.535, and $3{,}29 \times 10^{-6}$; (**C**) 0.467, 0.607, 0.394, and 9.62×10^{-4}; and (**D**) 0.467, 0.737, 0.588, and 4.32×10^{-5}, respectively.

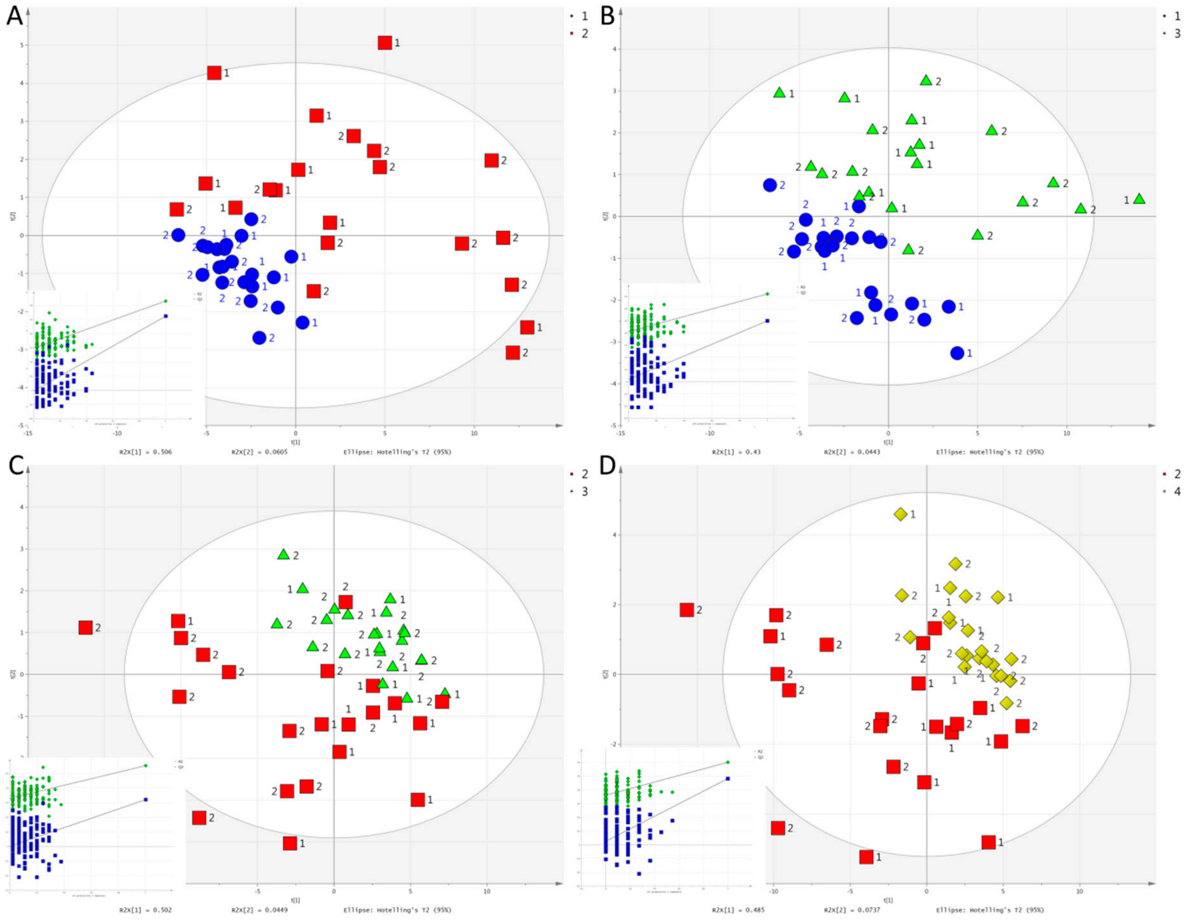

Figure S3. Score plots for the PLS-DA models of pair wise comparisons for HIIE: (**A**) 0 h (blue circles) vs. 2 h (red squares); (**B**) 0 h (blue circles) vs. 4 h (green triangles); (**C**) 2 h (red squares) vs. 4 h (green triangles); and (**D**) 2 h (red squares) vs. 24 h (yellow diamonds). Inserts are permutation plots. The MetS group is represented as 1 and the Healthy group as 2. R2X (cum), R2Y (cum), Q2Y (cum), and CV-ANOVA *p* value were: (**A**) 0.567, 0.631, 0.525, and 6.90×10^{-6}; (**B**) 0.474, 0.722, 0.501, and 2.70×10^{-5}; (**C**) 0.547, 0.554, 0.321, and 2.88×10^{-3}; and (**D**) 0.559, 0.602, 0.483, and 1.81×10^{-5}, respectively.

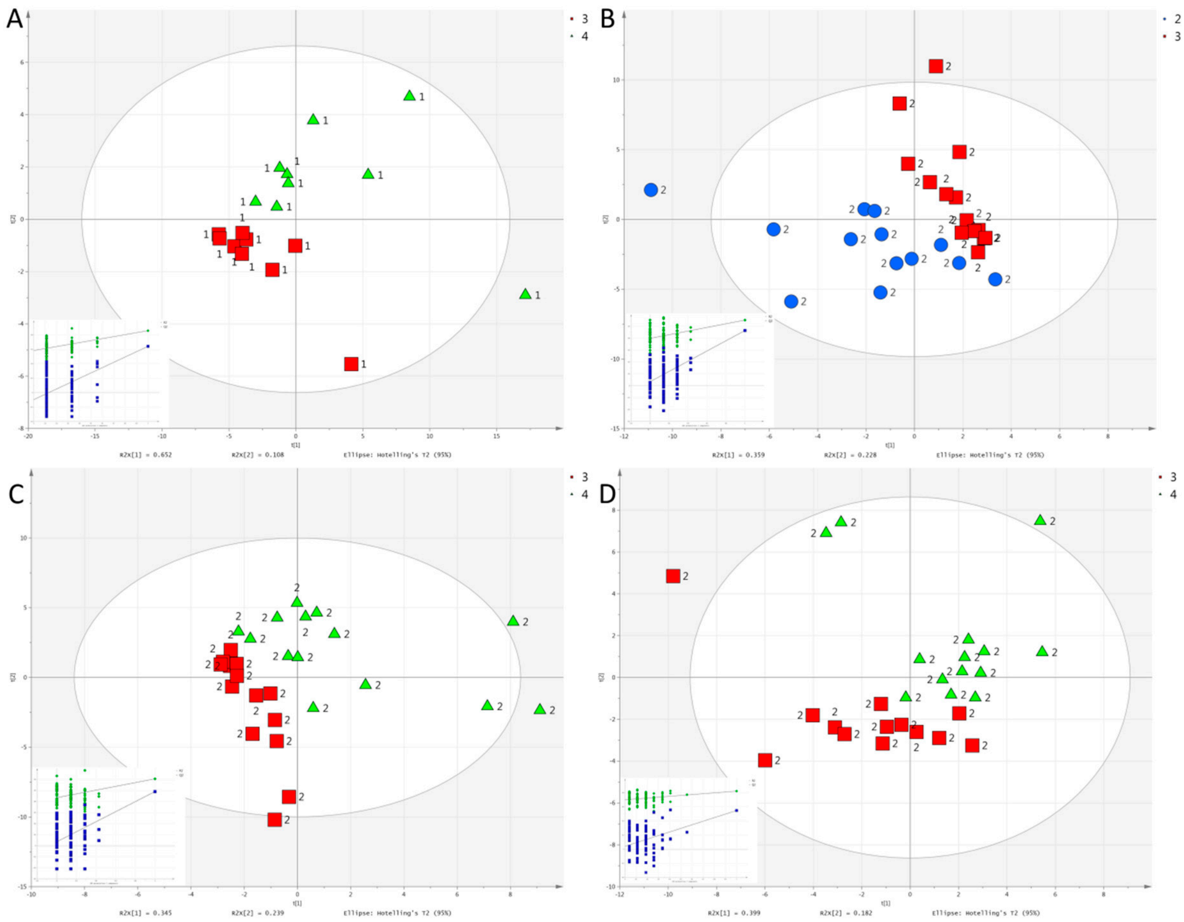

Figure S4. Score plots for the valid PLS-DA models of pair wise comparisons of exercise modes in each group separately: (**A**) MetS at 2 h, CME (red squares) vs. RE (green triangles); (**B**) Healthy at 2 h, HIIE (blue circles) vs. CME (red squares); (**C**) Healthy at 2 h, CME (red squares) vs. RE (green triangles); and (**D**) Healthy at 4 h, CME (red squares) vs. RE (green triangles). Inserts are permutation plots. The MetS group is represented as 1 and the Healthy group as 2. R2X (cum), R2Y (cum), Q2Y (cum), and CV-ANOVA *p* value were: (**A**) 0.813, 0.858, 0.641, and 4.08 × 10^{-2}; (**B**) 0.587, 0.552, 0.464, and 1.15 × 10^{-3}; (**C**) 0.584, 0.605, 0.490, and 1.53 × 10^{-3}; and (**D**) 0.617, 0.823, 0.548, and 2.07 × 10^{-3}, respectively.
